# Characterization of Affinity-Purified Isoforms of *Acinetobacter calcoaceticus* Y1 Glutathione Transferases

**DOI:** 10.1155/2014/750317

**Published:** 2014-04-24

**Authors:** Chin-Soon Chee, Irene Kit-Ping Tan, Zazali Alias

**Affiliations:** ^1^Institute of Biological Sciences, Faculty of Science, University of Malaya, 50603 Kuala Lumpur, Malaysia; ^2^Centre for Research in Biotechnology for Agriculture (CEBAR), University of Malaya, 50603 Kuala Lumpur, Malaysia

## Abstract

Glutathione transferases (GST) were purified from locally isolated bacteria, *Acinetobacter calcoaceticus* Y1, by glutathione-affinity chromatography and anion exchange, and their substrate specificities were investigated. SDS-polyacrylamide gel electrophoresis revealed that the purified GST resolved into a single band with a molecular weight (MW) of 23 kDa. 2-dimensional (2-D) gel electrophoresis showed the presence of two isoforms, GST1 (pI 4.5) and GST2 (pI 6.2) with identical MW. GST1 was reactive towards ethacrynic acid, hydrogen peroxide, 1-chloro-2,4-dinitrobenzene, and *trans,trans-hepta-2,4-dienal* while GST2 was active towards all substrates except hydrogen peroxide. This demonstrated that GST1 possessed peroxidase activity which was absent in GST2. This study also showed that only GST2 was able to conjugate GSH to isoproturon, a herbicide. GST1 and GST2 were suggested to be similar to F0KLY9 (putative glutathione S-transferase) and F0KKB0 (glutathione S-transferase III) of *Acinetobacter calcoaceticus* strain PHEA-2, respectively.

## 1. Introduction

Glutathione transferases (GSTs; EC2.5.1.18, formerly known as glutathione S-transferases) are a family of enzymes which catalyse the addition of the nucleophilic sulphur atom of glutathione (GSH) to the electrophilic centre of a large variety of hydrophobic molecules and thus make the conjugates more soluble and easily excreted from the cells. Apart from their catalytic role, GSTs are also capable of binding a number of endogenous and exogenous compounds noncatalytically. These include haem, bilirubin, hormones, flavonoids, fatty acids, and xenobiotics. GSTs have also been found to minimise the effects of oxygen toxicity and to mediate signal transduction during oxidative stress [1]. Therefore, GSTs are seen to play an important role in the detoxification of xenobiotics and endogenously toxic compounds.

GSTs are found in both eukaryotes and prokaryotes, and they can be divided into four families: cytosolic GST, mitochondrial GST, microsomal GST, and bacteria fosfomycin-resistant proteins [2]. In bacteria, four classes of cytosolic GSTs have been identified, namely, beta, chi, theta, and zeta. All beta class GSTs are characterized by the presence of cysteine residue in the active site that is responsible for the catalytic activity. The first GST of this class was purified from* Proteus mirabilis* [3]. The chi class is characterised by cyanobacterial GSTs of* Thermosynechococcus elongatus* BP-1 (TeGST) and* Synechococcus elongatus*  PCC6301 (SeGST) [4]. The theta class of GSTs is represented by dichloromethane dehalogenase produced by methylotrophic bacteria [5], while the zeta class is represented by tetrachlorohydroquinone (TCHQ) dehalogenase [6].

GSTs are found intracellularly and they have two general functions: to detoxify toxic compounds in the cells and to maintain cellular sulfhydryl groups in their reduced forms [7]. Reports have shown that bacterial GSTs are involved in many distinct metabolic processes such as biotransformation, degradation, and reductive dechlorination, which are linked to biodegradation of xenobiotics, defence against oxidative stress, protection against chemicals, and resistance towards antimicrobial drugs [8]. Bacterial GSTs are also involved in the degradation of several monocyclic aromatic compounds such as toluene, xylenes, phenols, and atrazine [9].

The detoxification ability of GSTs confers much importance to this group of enzymes in agriculture; for example, GSTs in arthropods and plant pests may be the cause of their resistance to insecticides and herbicides. Due to this advantage GSTs have been considered as potential candidate for the development of herbicide and stress tolerant transgenic plants [10]. GSTs are also seen as potential candidates for biotechnological applications in bioremediation and toxicology. Compared to the use of chemical or physical methods, bioremediation (the use of relevant microbes and/or enzymes) is often a more effective and safer alternative to clean up contaminated environments [8]. Biosensor development is another application whereby GSTs can be used as a tool to check for xenobiotic-contaminated environments and samples. Optical biosensors based on immobilised GSTs have been developed to detect captan [11], acrylamide [12], malathion [13], and atrazine [14]. These are all based on the ability of particular isoforms which are reactive towards specific xenobiotics. In view of the important properties and applications of GSTs, a continuous screening of bacteria for GSTs with new catalytic abilities to detoxify harmful or persistent chemicals is therefore important. This study describes the screening of soil bacteria for the production of GSTs, the purification of the enzymes by affinity chromatography, and some characterization to evaluate their catalytic abilities.

## 2. Materials and Methods

### 2.1. Chemicals

Unless otherwise stipulated, chemicals employed were of the highest grade obtainable. Dithiothreitol and phenylthiourea were from GE Healthcare. Centrifugal concentrators were obtained from Vivascience (Gottingen, Germany). Propoxur, isoproturon, fenoxaprop-ethyl, and clodinafop-propargyl were obtained from Sigma-Aldrich. Acrylamide, bisacrylamide, Biolyte ampholyte solution, and concentrated Coomassie Brilliant Blue protein reagent were obtained from BioRad Laboratories, Hercules, CA, USA. For isoelectric focusing, IPG strips were obtained from GE Healthcare. Buffer components were purchased from Sigma Chemical Co., St. Louis, USA.

### 2.2. Isolation, Screening, and Identification of GST-Expressing Bacteria

A quantity (1 g) of chemically contaminated soil collected from an illegal dump site located in University of Malaya, Kuala Lumpur, was placed in a sterile vial containing 10 mL of sterilized distilled water. The vial was shaken vigorously and left to stand overnight (18 hours) at 37°C in a water bath. The soil cultures were streaked on Nutrient Agar (Merck) plates and incubated for 18 hours at 37°C. Well separated colonies were purified on the same medium and maintained for screening for GST-expressing bacteria. Monochlorobimane (MCB) [15] was dissolved in acetonitrile at a concentration of 1 mg/mL (40 mM). Solutions were stored in a −20°C freezer and protected from light to avoid photolytic decomposition of MCB. To screen for GST-expressing bacteria, the MCB reagent was sprayed over each agar plate containing the bacterial isolates. Fluorescing colonies were visualized under long-wavelength (365 nm) UV light suggesting the presence of expressed GST. Isolates with intense fluorescence were grown, sedimented, and lysed (as mentioned in Section 2.4). The crude homogenate was tested for GST activity as described in Section 2.3. A positive isolate (with GST conjugating activity) was identified based on its 16S rRNA gene sequence.

### 2.3. Substrate Specificities

Enzymatic assays with 1-Chloro-2,4-dinitrobenzene (CDNB), ethacrynic acid (EA), sulfobromophthalein (BSP), p-nitrobenzyl chloride (NBC), trans-4-phenyl-3-buten-2-one (PBO) [16], and 3,4-Dichloronitrobenzene (DCNB) [17] were determined according to protocols described in the respective articles. Determination of glutathione peroxidase activity was performed by modifying the method described by Wendel [18]. Dichloromethane dehalogenase activity was determined using the method described by Sherratt et al. [19]. The ability to conjugate* trans,trans-*2,4-heptadienal was determined according to the method described by Brophy et al. [20].

### 2.4. Purification of Glutathione Transferases

A single colony of* Acinetobacter calcoaceticus* Y1 was picked and grown in nutrient broth (Oxoid) at 37°C for 18 hours after which the cells were pelleted by centrifugation (Beckman JA 7.5) at 3000 ×g for 20 minutes at 4°C. The cells were resuspended in 5 mL of 25 mM sodium phosphate buffer (pH 7.4), 1.0 mM EDTA, 0.1 mM DDT, 0.1 mM Phenylthiourea (PTU), and 0.1 mM phenylmethylsulfonyl fluoride (PMSF). The suspended cells were disrupted by sonication (Powersonic 603) for 20 minutes at 4°C and centrifuged (Beckman 80 Ti) at 100,000 ×g for 30 minutes at 4°C. The supernatant was applied onto the glutathione (GSH) agarose matrix (Sigma-Aldrich) packed in a Tricorn 5/20 column (GE Healthcare) which was connected to the AKTA purifier equipped with a fraction collector. The flow rate was set at 0.3 mL/min. The enzymes were eluted with buffer A containing 10 mM of GSH. These purified enzyme solutions were concentrated and either used immediately for kinetic and substrate specificity determination or were stored at −20°C until further analysis. The GST isoforms were separated using GSH-affinity chromatography and ion exchange chromatography columns arranged in tandem. GSH-agarose column with bound GSTs was connected to DEAE Sepharose fast flow column (1 mL) (GE Healthcare). The two connected columns were washed with five bed volumes of buffer B (25 mM sodium phosphate buffer, pH 6.0), and the enzymes were subsequently eluted with buffer B containing 10 mM of GSH and the flow-through was collected. Any GST that remained bound to the ion exchange column was eluted using buffer B containing 50 M NaCl.

### 2.5. Protein Determination

Protein concentration was determined using Coomassie Brilliant Blue R-250, and bovine serum albumin was used as the standard [21]. Protein standards were prepared in triplicate. Aliquots of bovine serum albumin (BSA) stock (1 mg/mL) and sample were pipetted into test tubes and the total volume was made 100 *μ*L. by addition of distilled water. To every sample and standard, 5 mL of Coomassie blue reagent was added, followed by vortexing. After 5 minutes the absorbance was read at 595 nm. The amounts of BSA in the standards were plotted against their average absorbance. The protein content of the samples was estimated from the standard curve.

### 2.6. 2-Dimensional Gel Electrophoresis and Isoelectrofocusing

Isoelectric focusing was conducted by using 2-D electrophoresis. The first dimension electrophoresis was carried out on a Multiphor II (GE Healthcare), and the second dimension electrophoresis was carried out in a Biorad Mini Protean II electrophoresis tank. Samples (the purified GST) for separation in the first dimension were mixed with 8 M urea, 2% CHAPS, 0.15% DTT, 30 mM thiourea, 2% Biolyte pH 3–10, and traces of Bromophenol Blue and were applied to a 7 cm, pH 3–10 IPG strip (Amersham Bioscience) during rehydration of the strip. The first dimension was set at a constant voltage of 200 V (1st stage, 1 min), followed by a gradient increase to 3500 V (2nd stage, 1 : 30 hr) and followed by constant 3500 V (3rd stage, 1 : 30 hr). Subsequently, proteins in the gel were reduced with DTT and alkylated with iodoacetamide. Electrophoresis in the second dimension was then run in 12% polyacrylamide gel in the presence of 0.1% SDS at 150 V. Sodium dodecyl sulfate polyacrylamide electrophoresis (SDS-PAGE) was performed according to the method of Laemmli [22].

To determine the pI of the sample protein, isoelectrofocusing was performed. The system used was Novex IEF Gels using the XCell* SureLock* Mini-Cell. The running buffers used were IEF Cathode Buffer (Invitrogen) and IEF Anode Buffer (Invitrogen). Protocols followed the manufacturer's recommendation. The running condition of the system was set at a constant voltage of 100 V for the first 1 hour, then 200 V for the next 1 hour and followed by 500 V for the last 30 minutes. Once the system stopped running, the plate was removed from the electrophoresis unit. The IEF gel was first fixed in 12% TCA for 30 minutes before staining with Coomassie Blue G-250 [23] and silver [24]. The stained gel was scanned using Image Scanner III (GE Healthcare) and visualized and analysed with Image Master Software.

### 2.7. In-Gel Digestion and Peptide Fingerprint Analysis

Protein spots (1 mm^3^) were excised and transferred into precleaned 0.5 mL capped tubes. The gels were destained two times by incubating in 200 *μ*L of 200 mM ammonium bicarbonate in 50% (v/v) acetonitrile for 45 minutes at 37°C [25]. The gel pieces were then dried using a CentriVap concentrator at room temperature for 10 minutes.

The dehydrated gel slices were rehydrated with 10 *μ*L of the trypsin solution (0.02 *μ*g/*μ*L) for one hour at room temperature. An additional 50 *μ*L of 40 mM ammonium bicarbonate in 10% (v/v) acetonitrile was added and incubated for 16 to 18 hours at 37°C. The extract was transferred into a new tube. The generated peptides were extracted from the gel pieces by treatment with 50 *μ*L 0.1% (v/v) trifluoroacetic acid twice for 45 minutes each at 37°C. The extracts were combined with the primary supernatant. The volume of the extract was reduced* in vacuo* to not more than 50% of total volume followed by fingerprinting analysis using MALDI-TOF (ABI 4800 PLUS). The protein was identified using Matrix Science MASCOT search engine.

### 2.8. Thin Layer Chromatography (TLC)

The conjugation of GSH to Propoxur, Isoproturon, Fenoxaprop-ethyl, and Clodinafop-propargyl was performed using the purified GST isoforms. All the reactions were performed in a volume of 3 mL at 25°C for 20 minutes. An aliquot of reaction mixtures were loaded on a 0.2 mm thick TLC silica gel plate (Merck) and developed using butanol/acetic acid/water (12 : 3 : 5; v/v/v) for 2 hours [26]. The air-dried plate was subsequently stained with ninhydrin (0.25%, w/v, in acetone). After 15 min, coloured spots were observed and marked.

## 3. Results and Discussion

**Figure 1:**
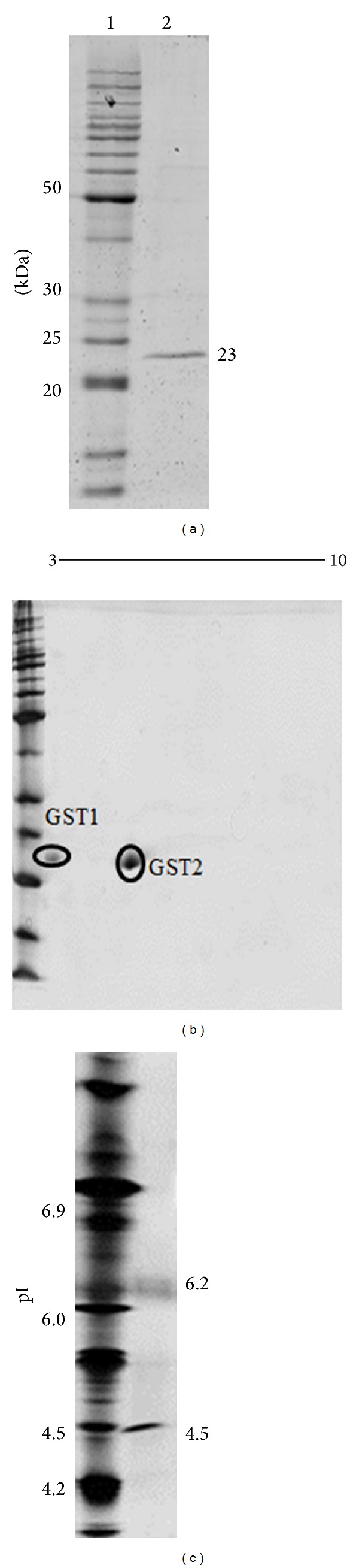
(a) SDS-PAGE of purified GSTs from* Acinetobacter calcoaceticus* Y1. Lane 1 shows the benchmark standard marker (Invitrogen). Lane 2 shows the presence of single band GST with MW estimated at 23 kDa (10 *μ*g). (b) 2-D gel electrophoresis (IPG 3–10) of purified GSTs resolved into two isoforms (30 *μ*g). (c) Isoelectric-focusing of the purified GST (10 *μ*g). The calculated pIs are 4.5 and 6.2 for GST1 and GST2, respectively. SERVA IEF marker (Invitrogen) was used for pI estimation. Gels were stained with colloidal coomassie blue (a and b) and silver (c).

The screening of GST-expressing bacteria from soil is possible by using MCB [5] as shown in this study. The 16S rRNA gene is highly conserved in bacteria; therefore its sequence is commonly used to identify bacterial isolates [27]. By analysing its 16S rRNA gene sequence, the GST-expressing bacteria isolated in this study were identified and named as* Acinetobacter calcoaceticus *Y1. Crude activity on EA conjugation was detected from the lysed culture of this isolate, confirming the presence of GSTs in the cells. A single band protein was visualized on SDS-PAGE after purification using GSH-affinity chromatography. The molecular weight (MW) was estimated to be 23 kDa (Figure 1(a)) which was within the range of GSTs molecular weights. A further 2-D gel analysis revealed that the band consisted of two isoforms of GSTs (Figure 1(b)) estimated by vertical isoelectric focusing to have pI values of 4.5 and 6.2, respectively (Figure 1(c)). The presence of multiple isoforms of GSTs is rarely documented in bacteria compared to insects or human. The functions of both isoforms are of great interest; therefore we attempted to purify and characterize each isoform.

Both isoforms were separated by using CM and DEAE Sepharose fast flow columns (GE Healthcare). Only “flow-through” was collected from both columns and the eluate was discarded. When the CM Sepharose column was used, isoform pI 4.5 would come out in the “flow-through” while isoform pI 6.2 would appear in the second eluate generated by 10 mM of sodium chloride (NaCl). On the other hand, when the DEAE Sepharose column was used, isoform pI 6.2 would come out in the “flow-through” while isoform pI 4.5 would appear in the second eluate generated by 10 mM of sodium chloride (NaCl). However, neither isoform pI 6.2 nor isoform pI 4.5 from the second eluate fractions showed any GSH-conjugation activity. There was absence of band when both isoforms were run on SDS-PAGE (data not shown). We suggest that the isoforms which were eluted from both columns using NaCl solution might have experienced denaturation as a result of distortion of the ionic interaction within the protein. According to Date and Dominy [28], salts such as NaCl might influence protein stability through electrostatic mechanisms as well as through nonpolar Hofmeister effect. For convenience of discussion isoform pI 4.5 and isoform pI 6.2 were designated as GST1 and GST2, respectively.

GST1 showed a single band on SDS-PAGE which was consistent with the result shown in Figure 1(a) where a single band also occurred at 23 kDa. On the other hand, GST2 displayed different physical characteristic compared to GST1. GST2 tended to form aggregation of which a single band at estimated 110–115 kDa was observed instead of at 23 kDa. Extracellular aggregation of GSTs had not been reported so far but intracellular aggregation of GSTs had been reported for human Pi (*π*) class GST (hGSTP) [29] and* Onchocerca volvulus* GST (OvGST) [30]. Intracellular protein aggregation is mainly caused by posttranslational modification of protein through the process of glycosylation and phosphorylation.

**Figure 2:**
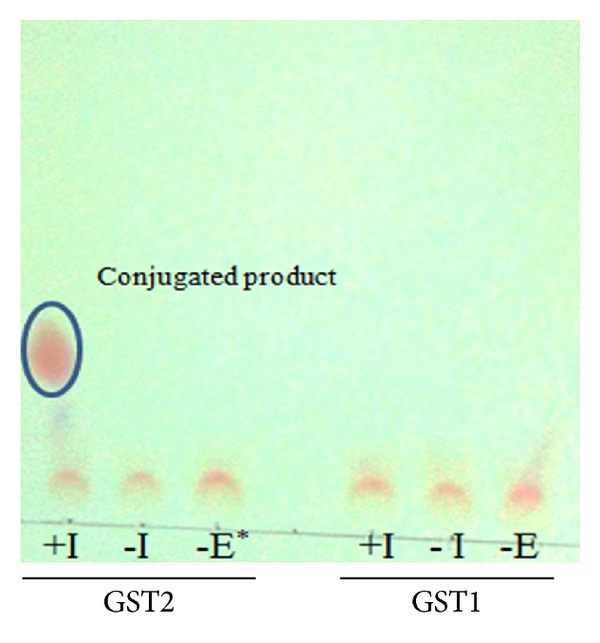
Thin layer chromatography indicating conjugation of isoproturon by GST2 (isoform pI 6.2) (circled spot). No conjugated product was seen when GST1 (isoform pI 4.5) was tested. +I = with isoproturon, −I = without isoproturon, −E = without enzyme.

Table 1 indicates that both GST1 and GST2 had highest affinity towards ethacrynic acid compared to the other substrates. This was an early indication that both isoforms behave similar to a Pi (*π*) class GSTs [31]. Although 1-Chloro-2,4-dinitrobenzene is a common substrate for GSTs from other organisms, both isoforms from the* A. calcoaceticus* Y1 isolated in this study recorded a considerably low activity towards the substrate. This characteristic is in line with bacterial GSTs. Our study showed that GST1 reacted with hydrogen peroxide, indicating that it is a selenium-dependent glutathione peroxidase [32]. In contrast, GST2 was not shown to be catalytically active towards hydrogen peroxide and cumene hydroperoxides. The conjugation of both isoforms with a lipid peroxidation product such as* trans,trans-*hepta-2,4-dienal also suggested that both the isoforms may play a role in combating oxidative stress due to lipid peroxidation. No dichloromethane dehalogenase activity was seen for both isoforms, implying that they were not of the theta class GST. Inference had been made on the ability of GSTs to degrade morpholine [33]. In this study, the ability of both isoforms to conjugate pesticides was also investigated. Our findings showed that both were unable to conjugate propoxur, fenoxaprop-ethyl, and clodinafop-propargyl. However, GST2 was able to conjugate isoproturon (Figure 2), a feature not seen with GST1. Allocati et al. [8] had reported that bacterial GSTs were able to detoxify several classes of herbicides. Early studies have linked conjugating activity with DCNB with insecticide resistance, particularly in insects. However, later studies showed that this was not necessarily correlated; for example, an epsilon class of GST in fruit flies, CG16936, [34] and in* Anopheles gambiae*, GSTE1-1z, [35] had conjugating activity towards DCNB but did not confer insecticide resistance. Our findings showed that GST2 had no activity with DCNB but was able to selectively conjugate isoproturon, suggesting its specific functional role in the cells. The substrate specificities of both isoforms were different and thus it is believed that they were of two existing homodimers.

Due to lack of bacterial genome databases, we were unable to significantly identify each isoform as glutathione transferase through MALDI-TOF mass spectrometry analysis. The putative glutathione transferases of* Acinetobacter calcoaceticus* strain PHEA-2 [36] which were, however, retrieved from http://www.uniprot.org/uniprot/ are tabulated in Table 2. It shows that the strain has 10 putative GSTs of which two of the expressed proteins have close characteristics to GST1 and GST2. As shown in Table 2, The UniProt identifiers of F0KLY9 (putative glutathione S-transferase) and F0KKB0 (Glutathione S-transferase III) are similar in MW and pI values to GST1 and GST2, respectively. Nevertheless, more confirmative evaluation is warranted for the identification of the isoforms, such as N-terminal sequencing.

An amino acid sequence similarity check indicated that both isoforms (F0KLY9 and F0KKB0) have no similarity to any class of GSTs in the database. In most GST classes the N-terminal tyrosine residue is the active residue that interacts with GSH to stabilise the thiolate anion. In theta and zeta classes the role is carried out by serine residue, while in omega and beta classes cysteine is the active residue [2], at most of the time in positions 5 and 6.

In Table 3, the retrieved amino acid sequence of F0KLY9 (putative glutathione S-transferase) indicates the presence of tyrosine residue at position 6. However, none of the abovementioned amino acids known to be active residues was found in the N-terminal sequence of F0KKB0 (Glutathione S-transferase III). Besides the N-terminal amino acids, C-terminal histidine-106 and lysine-107 had been reported to contribute to the interaction with GSH [37]. The same behaviour was seen in* Drosophila melanogaster* GSTD3 where distal amino acids that are catalytically essential for conjugation (unpublished data from our laboratory). Therefore, to identify the catalytically active residues in F0KKB0 (Glutathione S-transferase III) is of a great interest. Both putative GSTs were however not identified to any class of GSTs when submitted to BLAST (http://blast.ncbi.nlm.nih.gov/).

## 4. Conclusion

A locally isolated bacterium, identified as* Acinetobacter calcoaceticus* Y1, was found to produce a GST with a molecular weight of 23 kDa (based on SDS-PAGE). Through the use of two-dimensional chromatography, the band was found to compose of two isoforms. Isoelectric focusing determined that the isoforms, named GST1 and GST2, have pI values of 4.5 and 6.2, respectively. Both isoforms were able to conjugate with substrates involved in lipid peroxidation, and GST1 had shown peroxidase activity. These suggest that GST1 and GST2 may have roles in combating oxidative stress. GST2 was shown to specifically conjugate isoproturon. The behaviour could protect the bacteria against harmful toxic herbicide. Both GST1 and GST2 closely resemble putative GSTs of* Acinetobacter calcoaceticus* (strain PHEA-2), F0KLY9 (Putative glutathione S-transferase) and F0KKB0 (Glutathione S-transferase III), respectively. The findings of this study concur that GST-expressing bacteria are found in the soil and that they might involve in the detoxification and degradation of xenobiotics including pesticides. Further studies on bacterial GSTs are warranted because of the potential of soil bacteria as natural and ecofriendly agents in environmental bioremediation. GSTs alone would however not be sufficiently effective for a broad-based xenobiotic biotransformation, perhaps engineered fusion proteins with several distinct catalytic capabilities should be one of the promising approaches. All in all, GSTs and their genes are promising tools to develop applications in biological treatment of environmental and industrial pollutants.

## Figures and Tables

**Table 1:** Specific activities of GST1 and GST2 towards selected substrates.

Substrate	Specific activity (*μ*mol/min/mg)	
	GST1	GST2
Ethacrynic acid	24.91 ± 1.24	15.71 ± 2.53
Hydrogen peroxide	3.93 ± 0.38	nd
1-Chloro-2,4-dinitrobenzene	0.61 ± 0.122	0.542 ± 0.127
*trans,trans-*Hepta-2,4-dienal	0.11 ± 0.02	0.084 ± 0.01
1,2-Dichloro-4-nitrobenzene	Nd	nd
*p*-nitrophenyl chloride	Nd	nd
Sulfobromophthalein	Nd	nd
*trans-*4-Phenyl-3-butene-2-one	Nd	nd
Cumene hydroperoxide	Nd	nd
Dichloromethane	Nd	nd

nd: not detected.

**Table 2:** List of putative glutathione transferases of *Acinetobacter calcoaceticus* (strain PHEA-2) retrieved from http://www.uniprot.org/uniprot/. pI and MW values were computed at Compute pI/Mw (http://www.expasy.org/tools/). Bold indicates that F0KLY9 (putative glutathione S-transferase) and F0KKB0 (glutathione S-transferase III) were similar in MW and pI values to GST1 and GST2, respectively.

UniProt identifiers	Protein name	Gene name	a.a.* length	pI/MW
F0KI99	Putative glutathione S-transferase	gstB BDGL_000451	203	4.91/22987.64
**F0KLY9**	**Putative glutathione S-transferase**	**erd13 BDGL_003315**	**201**	4.85/22385.40
**F0KKB0**	**Glutathione S-transferase III**	**gst3 BDGL_000699**	**214**	5.97/24579.78
F0KI95	Putative glutathione S-transferase	yliJ BDGL_000447	208	5.19/24435.28
F0KGP1	Glutathione transferase FosA	fosA BDGL_002726	135	5.34/15740.79
F0KH55	Putative glutathione S-transferase	BDGL_001572	228	6.01/26244.78
F0KL12	Glutathione S-transferase-like protein	yghU BDGL_000797	282	5.62/31834.09
F0KLP2	Glutathione S-transferase	BDGL_000872	246	5.52/28865.77
F0KND6	Putative glutathione S-transferase	yfcG BDGL_003500	206	5.13/23912.28
F0KJF2	Glutathione S-transferase	gst3 BDGL_003061	222	6.79/26004.25

*a.a.: amino acid.

**Table 3:** Protein (FASTA) sequences for F0KLY9 (putative glutathione S-transferase) and F0KKB0 (glutathione S-transferase III) were retrieved from http://www.uniprot.org/uniprot/. Bold and italic is the identified active residue (Y, S, or C at positions 5 or 6) which is present in F0KLY9 (putative glutathione S-transferase) amino acid sequence.

UniProt Identifiers	Amino acid sequences
F0KLY9	MSLKL***Y***TNKESRGVVIDWLLVELGVECERIEVAYKTEMKSPEYLKLN PFGKVPVLVDGDVVIYELGAICAYLADKFSDKGLAPALDDPKRGLYYR WLFLMAGPWEAAGVDKALGIEVSPEQKMFVGYGDYNDAYQALVQGL SEANPYVCGEQFTAADVSVGAMLLWQLKMNAIESHPAITRYVETIKQR EGLKQSTMGQLL
F0KKB0	MSIILHHLNASRSFRILWLLEEINQPYELKSYFRDKTTNLAPQELKNIHP LGKSPVIELNGKVIAESGAIVEILIEKFAPQLMPAKDSDSYLDYLQWVH FSESSAMVPYLLNIFNSIELKNGTQLKFLDQYAHTELDKVFSYLDQQLV GKKFLVGNSLTGADFMIGFVVYGLINSLNIRSKYLNIEQYVKSLENLES WQKAMSIEQNLHHQTNA
